# Association Between the Surrogate Markers of Insulin Resistance and Chronic Kidney Disease in Chinese Hypertensive Patients

**DOI:** 10.3389/fmed.2022.831648

**Published:** 2022-02-07

**Authors:** Yumeng Shi, Lihua Hu, Minghui Li, Wei Zhou, Tao Wang, Lingjuan Zhu, Huihui Bao, Xiaoshu Cheng, Ping Li

**Affiliations:** ^1^Department of Cardiovascular Medicine, The Second Affiliated Hospital of Nanchang University, Nanchang, China; ^2^Jiangxi Provincial Cardiovascular Disease Clinical Medical Research Center, Nanchang, China; ^3^Department of Cardiovascular Medicine, Peking University First Hospital, Beijing, China; ^4^Department of Cardiovascular Medicine, Inner Mongolia People's Hospital, Hohhot, China; ^5^Center for Prevention and Treatment of Cardiovascular Diseases, The Second Affiliated Hospital of Nanchang University, Nanchang, China

**Keywords:** chronic kidney disease, lipid accumulation product index, triglyceride-glucose index, triglycerides to high-density lipoprotein cholesterol ratio, visceral adiposity index

## Abstract

**Background:**

We aim to evaluate the four surrogate markers of insulin resistance (IR), including triglyceride-glucose index (TyG), lipid accumulation product index (LAP), visceral adiposity index (VAI), triglycerides to high-density lipoprotein cholesterol ratio (TG/HDL), on prevalence of chronic kidney disease (CKD) and to examine any possible effect modifiers in Chinese hypertensive patients.

**Methods:**

A total of 13,055 hypertensive participants were included in this cross-sectional study. In addition, average age of the study population was 63.81 ± 9.46 years, and 47.66% of them are men. The primary outcome was CKD, defined as eGFR <60 ml/min/1.73 m^2^. Multivariate logistic regression analysis and the generalized additive model and a fitted smoothing curve (penalized spline method) were used to examine the association between the surrogate markers of IR and CKD.

**Results:**

Four surrogate markers of IR were independently and positively associated with CKD in a dose-response fashion. The association between four surrogate markers of IR and the prevalence of CKD was examined as a continuous variable per one unit increment and also as a categorical variable using tertiles with the tertiles (T1) as the reference group. In the fully adjusted model, multivariate logistic analyses showed that the per one unit increments of the TyG, LAP, VAI, and TG/HDL ratios were all significantly associated with 42, 31, 67, and 78% higher risk for CKD, respectively. Consistently, the adjusted ORs (95% CI) for CKD were 1.48 (1.21, 1.81), 1.34 (1.06, 1.69), 1.26 (1.03, 1.53), 1.35 (1.12, 1.63) when comparing the highest tertile to the lowest tertile of the TyG, LAP, VAI, and TG/HDL ratios, respectively. The stratification analysis showed that a significant positive correlation between TyG, VAI, and TG/HDL and CKD in patients over 65 years old.

**Conclusion:**

Four surrogate markers of IR were independently and positively correlated with CKD, and LAP was better than the other surrogate markers of IR for predicting CKD. Only among participants aged over 65 years were higher levels of TyG, VAI and TG/HDL found to be closely related to the increased prevalence of CKD.

## Introduction

Chronic kidney disease (CKD), as a major global public health problem, has aroused widespread concern (1–5). The burden of CKD is not limited to the need for renal replacement therapy for end-stage renal disease (ESRD), and cardiovascular events and mortality are strongly affected by renal involvement (6, 7). It is estimated that five million to 10 million people worldwide die annually from kidney diseases such as CKD, renal failure and ESRD (8, 9). In China, the prevalence of CKD is increasing rapidly due to rising risk factors such as diabetes, hypertension, unhealthy diet, inappropriate physical activity and metabolic syndrome (10–12). In addition, compared with other risk factors, the development of nondiabetic CKD has been confirmed to be closely related to hypertension (13). If patients with hypertension are complicated with CKD at the same time, their cardiovascular disability rate and death risk will greatly increase (14). Therefore, a better understanding of the potential risk factors of CKD increase in hypertensive patients may help to prevent CKD and related cardiovascular diseases. Recently, more and more scholars pay attention to the relationship between insulin resistance (IR) and CKD. and put forward that IR can predict the risk of CKD (15–17).

IR is a pathological state in which tissues have a decreased sensitivity to insulin, leading to a compensatory rise in circulating insulin to maintain normal blood glucose levels (18, 19). The gold standard for evaluating IR is the hyperinsulinemic-normoglycemic clamp test (20). However, the hyperinsulinemic-normoglycemic clamp test is rarely performed in the epidemiological investigations of large sample populations because it requires special equipment, which is time-consuming and expensive. Therefore, to determine whether insulin resistance exists in epidemiological investigations, many researchers have developed simple and feasible alternative markers of IR, such as the triglyceride glucose index (TyG) (21), lipid accumulation product index (LAP) (22), visceral adiposity index (VAI) (23) and TG/HDL ratio (24). The majority of studies have studied the risk of CKD caused by single surrogate markers of IR, such as TyG (25), LAP (26), VAI (27–29) and the TG/HDL ratio (30). However, published data on the relationship between all surrogate markers of IR and CKD risk are limited.

The objective of the present study was to investigate whether the four surrogate markers of IR are associated with the prevalence of CKD and to examine any possible effect modifiers in Chinese hypertensive patients based on data from a large observational study, the China H-type Hypertension Registry Study (CHHRS).

## Methods

The research program was approved by the Ethics Commission of Anhui Medical University's Institute of Biomedicine (No. CH1059). Written informed consent was formally obtained from all participants.

### Study Population

The data are from the CHHRS Study (Registration number: ChiCTR1800017274) in rural areas of southern China. Briefly, CHHRS is an ongoing real-world, observational registry study conducted from March 2018 to August 2018 in Wuyuan, Jiangxi Province, China. The inclusion criterion was hypertensive patients over 18 years old, The exclusion criteria were as follows: (1) unable to sign informed consent due to psychological or nervous system damage and (2) inability to be followed up due to a relocation plan in the short term. The details on the trial design and methods have been described in previous publications (31).

**Figure 1:**
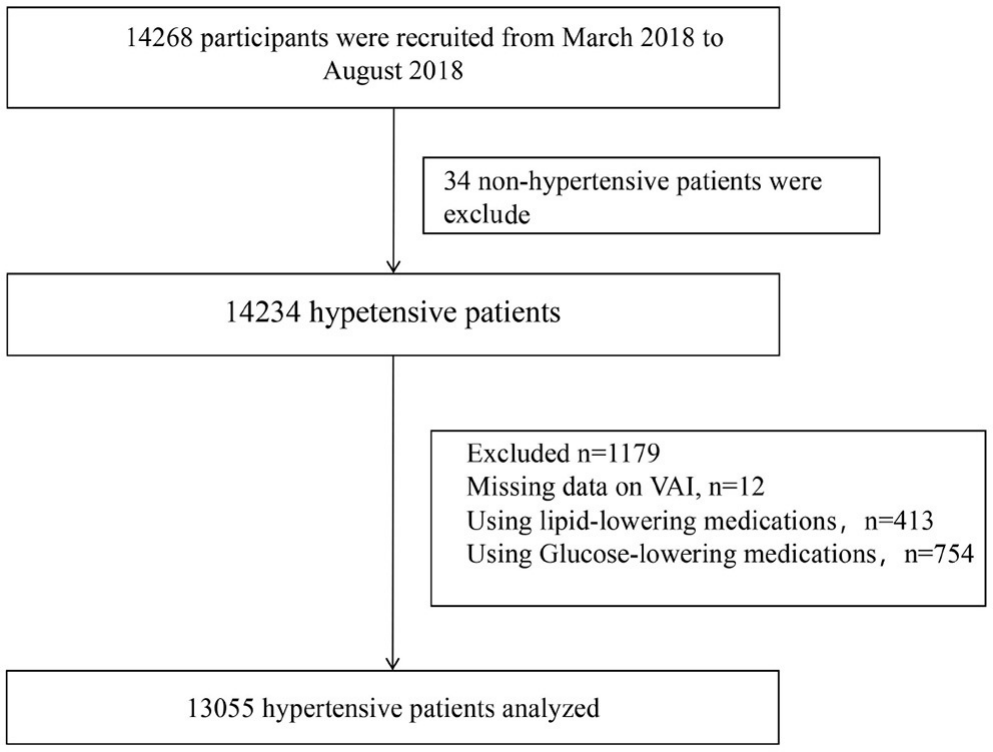
Flow chart of study participants.

Baseline data collection was completed by 14268 participants. After excluding subjects with nonhypertension (*n* = 34), missing VAI data (*n* = 12), using glucose-lowering medications (*n* = 754) and using lipid-lowering medications (*n* = 413), 13,055 participants were analyzed in the current study (Figure 1).

### Data Collection

With the help of trained researchers, all participants completed a questionnaire survey on socioeconomic and demographic factors, lifestyle habits, history of disease, and drugs used in the past 2 weeks. In this standardized questionnaire, smoking habits and drinking habits were classified variables, and their classifications were never and present, respectively. The education level was classified according to less than junior college or at least junior college. According to the participants' personal evaluation, physical activities were classified as mild, moderate or vigorous.

At the same time, the baseline data also included anthropometric indicators such as height, weight and waist circumference (WC). Weight and height were measured to the nearest 0.1 kg and 0.1 cm, respectively, with the participants wearing lightweight clothing and without shoes. WC was measured to the nearest 5 mm directly touching the participant's skin using cloth tape. Body mass index (BMI), in kg/m^2^, was calculated as the weight divided by the height squared. After resting for at least 10 min, an electronic sphygmomanometer (Omron; Dalian, China) was used to measure blood pressure (BP) in the sitting position, and the average of the three blood pressures was used in the final analysis. Hypertension was defined as seated resting systolic blood pressure (SBP) ≥ 140 mm Hg or diastolic blood pressure (DBP) ≥ 90 mm Hg at screening visits or on the use of antihypertensive medications.

After 12 h of fasting, blood samples were collected from the anterior cubital vein between 8 and 10 a.m. Homocysteine (Hcy), fasting glucose, serum lipid [total cholesterol (TC), triglyceride (TG), low-density lipoprotein cholesterol (LDL-C), high-density lipoprotein cholesterol (HDL-C)], serum uric acid, serum creatinine, aspartate aminotransferase (AST), alanine aminotransferase (ALT), and serum γ-glutamyltransferase (GGT) were assayed in a single laboratory (Biaojia Biotechnology Laboratory, Shenzhen, China) on an automatic clinical analyzer (Beckman Coulter). The Chronic Kidney Disease Epidemiology Collaboration (CKD-EPI) equation (32) was used to calculate the estimated glomerular filtration rate (eGFR). The formulas for eGFR are as follows: males with creatinine ≤ 0.9 mg/dl: eGFR (ml/min/1.73 m^2^) = 141 × (creatinine/0.9)^−0.411^ × 0.933^age^; males with creatinine > 0.9 mg/dl: eGFR (ml/min/1.73 m^2^) = 141 × (creatinine/0.9)^−1.209^ × 0.933^age^; females with creatinine ≤ 0.7 mg/dl: eGFR (ml/min/1.73 m^2^) = 144 × (creatinine/0.7)^−0.329^ × 0.933^age^; and females with creatinine > 0.7 mg/dl: eGFR (ml/min/1.73 m^2^) = 144 × (creatinine/0.7)^−1.209^ × 0.933 ^age^.

### Definition of the TyG, LAP,VAI, TG/HDL, and CKD

The TyG index was calculated as ln[fasting triglycerides (mg/dl) × fasting glucose (mg/dl)/2] (33). LAP was determined from WC (cm) plus TG (mmol/L) for men [(WC-65) × TG] and women [(WC-58) × TG] (34). VAI was calculated using the following sex-specific equations, where the units for WC, BMI, and TG and HDL are cm kg/m^2^, and mmol/L: males: VAI = [WC/[39.68 + (1.88 × BMI)]) × (TG/1.03) × (1.31/HDL); females: VAI = [WC/[36.58 + (1.89 × BMI)]) × (TG/0.81) × (1.52/HDL) (35). The TG/HDL ratios were calculated as TG divided by HDL. The diagnostic criteria for CKD were eGFR <60 ml/min/1.73 m^2^ (36).

### Statistical Analysis

*P* < 0.05 with two tails was statistically significant. All statistical analyses were performed using the statistical package R (http://www.R-project.org, The R Foundation) and Empower Stats (http://www.empowerstats.com, X&Y Solutions, Inc., Boston, MA).

Continuous variables are presented as the means ± standard deviations (SDs) or medians (interquartile ranges) (IQRs), and categorical variables are presented as numbers (percentages). A *t*-test of students' normally distributed data, the Mann–Whitney test of nonparametric nonnormally distributed data and χ^2^ test of classified data were used to compare the differences in data characteristics with or without CKD.

Because LAP, VAI and TG/HDL had skewed distributions, they were transformed into normal distributions by lg-transformation. If the estimated effect changed by ≥10% alone, the variables called traditional risk factors and potential confounding factors of chronic kidney disease were selected (37). Logistic regression analyses were performed to assess the association between four surrogate markers of IR (TyG, LAP, VAI and the TG/HDL-C ratios) and CKD. The association between four surrogate markers of IR and the prevalence of CKD was examined as a continuous variable per one increment and as a categorical variable using tertiles with the tertiles (T1) as the reference group. In the case of the logistic regression analysis of the ORs of CKD, the three models constructed were as follows: Model 1 was adjusted for age and sex; Model 2 was adjusted for age, sex, BMI, education, and physical activity; and Model 3 was adjusted for age, sex, BMI, education, physical activity, current smoking, current drinking, SBP, DBP, pulse, Hcy, AST, GGT, LDL-C, antihypertensive drugs, antiplatelet drugs, and self-reported diabetes. To examine the significant associations between four surrogate markers of insulin resistance (IR) and CKD, a generalized additive model and a fitted smoothing curve (penalized spline method) were used to further explore the shape of their dose–response relations.

Tests for interaction were performed using a likelihood ratio test to compare models with and without interaction terms. Further stratified analyses by subgroups, including sex (male or female), age (<65 or ≥65 years), BMI (<25 or ≥25 kg/m^2^), current smoking (no or yes), current drinking (no or yes), physical activity (mild, moderate, vigorous), serum LDL-C (<2.6 or ≥2.6 mmol/L), SBP (<140, 140–159, ≥160 mm Hg), and self-reported diabetes (no or yes), were also explored by multivariable logistic regression models to test for consistency of results.

## Results

### Baseline Characteristics

A total of 13,055 patients with hypertension were enrolled in the study. In addition, the average age of the study population was 63.81 ± 9.46 years, and 47.66% of them were men. There were 1,218 (9.32%) participants with CKD and 1,775 (13.60%) participants with diabetes.

According to CKD, the clinical and demographic characteristics of the participants are presented in Table 1. Participants with CKD were older and more often male with self-reported diabetes; they had higher levels of Hcy and lower levels of physical activity, BMI, DBP, LDL-C, ALT, LAP, and VAI. They were also less frequently current drinkers and more frequently using antihypertensive drugs and antiplatelet drugs (all *P* < 0.05). There was no significant difference in smoking, education, SBP, GGT, pulse, TyG, AST, or TG/HDL ratio among patients with or without CKD (all *P* > 0.05).

**Table 1:** Baseline characteristics of study participants^a^.

**Variable**	**Total**	**Non-CKD**	**CKD**	***P*** **-value**
N	13,055	11,837	1,218	
Age, y	63.81 ± 9.46	63.07 ± 9.18	70.97 ± 9.18	<0.001
Male, *n* (%)	6,222 (47.66%)	5,530 (46.72%)	692 (56.81%)	<0.001
Current smoker, *n* (%)	3,448 (26.42%)	3,117 (26.34%)	331 (27.20%)	0.517
Current drinker, *n* (%)	2,923 (22.40%)	2,742 (23.17%)	181 (14.87%)	<0.001
Education				0.499
Less than high school	10,456 (98.54%)	9,335 (98.51%)	1,121 (98.77%)	
At least high school	155 (1.46%)	141 (1.49%)	14 (1.23%)	
Physical activity				<0.001
Mild	5,879 (55.40%)	5,088 (53.69%)	791 (69.69%)	
Moderate	2,500 (23.56%)	2,322 (24.50%)	178 (15.68%)	
Vigorous	2,232 (21.03%)	2,066 (21.80%)	166 (14.63%)	
BMI, kg/m^2^	23.50 ± 3.75	23.60 ± 3.76	22.58 ± 3.53	<0.001
SBP, mmHg	148.56 ± 17.84	148.47 ± 17.50	149.50 ± 20.89	0.055
DBP, mmHg	89.17 ± 10.80	89.49 ± 10.59	86.02 ± 12.20	<0.001
pulse, bpm	76.51 ± 14.16	76.46 ± 13.94	77.00 ± 16.19	0.202
Hcy,μmol/L	15.02 (12.49–19.17)	14.58 (12.31–18.10)	21.77 (17.91–28.15)	<0.001
LDL-C, mmol/L	3.00 ± 0.80	3.00 ± 0.79	2.92 ± 0.86	0.001
AST, U/L	24.00 (20.00–30.00)	24.00 (20.00–30.00)	24.00 (20.00–30.00)	0.980
ALT,U/L	16.00 (12.00–23.00)	17.00 (12.00–24.00)	15.00 (11.00–21.00)	<0.001
GGT, U/L	21.00 (15.00–34.00)	21.00 (15.00–34.00)	21.00 (15.00–32.00)	0.272
TyG	8.87 ± 0.61	8.88 ± 0.61	8.86 ± 0.61	0.387
LAP	32.66 (16.80–56.60)	33.20 (17.28–57.12)	27.26 (12.48–51.71)	<0.001
VAI	1.54 (0.93–2.55)	1.55 (0.93–2.56)	1.45 (0.88–2.44)	0.024
TG/HDL ratio	0.95 (0.61–1.53)	0.95 (0.61–1.53)	0.94 (0.62–1.50)	0.681
Antihypertensive drugs, *n* (%)	8,272 (63.38%)	7,362 (62.21%)	910 (74.77%)	<0.001
Antiplatelet drugs, *n* (%)	271 (2.08%)	234 (1.98%)	37 (3.04%)	0.013
^$^Self-reported diabetes, *n* (%)	1,775 (13.60%)	1,575 (13.31%)	200 (16.42%)	0.003

a *Values are mean ± SD, median [IQR] for skewed variables, or n (%) for categorical variables*.

*BMI, body mass index; SBP, systolic blood pressure; DBP, diastolic blood pressure; Hcy, homocysteine; LDL-C, low-density lipoprotein cholesterol; AST, aspartate aminotransferase; ALT, alanine aminotransferase; GGT, Serum γ-glutamyltransferase, U/L*.

$ *Self-reported diabetes was defined as self-reported physician diagnosis of diabetes or FBG concentration ≥7.0 mmol/L or use of glucose-lowering drugs*.

### Association of Surrogate Markers of Insulin Resistance With CKD

**Figure 2:**
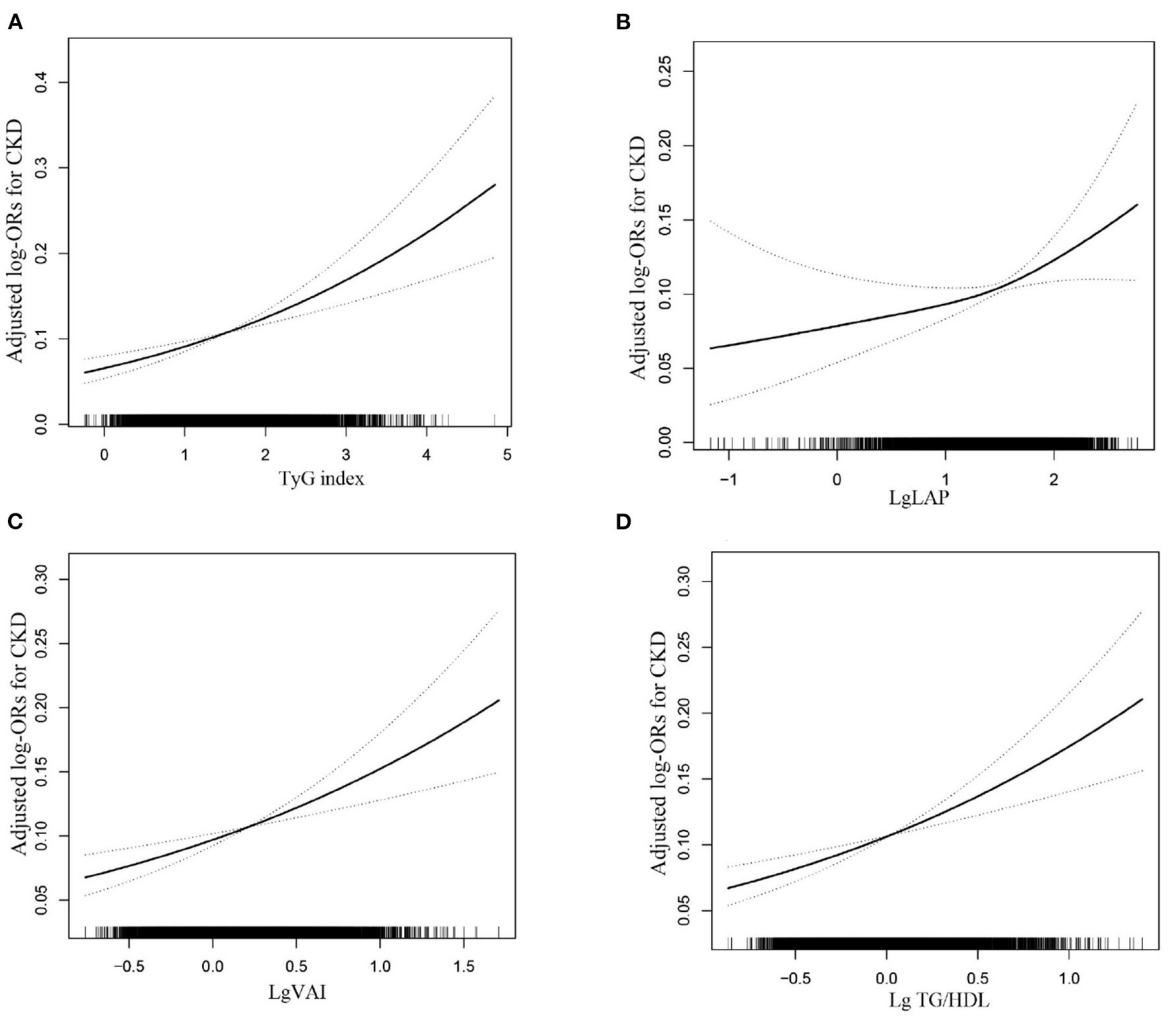
The association between the TyG **(A)**, LAP **(B)** and VAI **(C)** and TG/HDL **(D)** level and the risk of CKD. The solid line and dashed line represent the estimated values and their corresponding 95% confidence interval, respectively. The adjustment factors included age, sex, BMI, education, physical activity, current smoking, current drinking, SBP, DBP, pulse, Hcy, AST, GGT, LDL-C, Antihypertensive drugs, Antiplatelet drugs, self-reported diabetes.

Multiple logistic regression analyses were used to assess the association between surrogate markers of IR and CKD. Table 2 shows that the ORs and 95% CIs for CKD showed a significant gradual increase at higher levels of TyG, LAP, VAI and TG/HDL in a dose-dependent manner (all *P* for trend < 0.05). The per one increment of the TyG, LAP, VAI and TG/HDL levels was significantly associated with 42, 31, 67 and 78% higher risks for CKD, respectively. After full adjustment, hypertension subjects in the top tertiles of TyG had 1.48-fold increased odds of prevalent CKD relative to those in the bottom tertiles of TyG (OR: 1.48, 95% CI: 1.21–1.81), and the ORs of prevalent CKD increased by a factor of 1.34 for individuals in the highest tertiles of the LAP group (OR: 1.34, 95% CI: 1.06–1.69) compared with the reference group. Compared with the reference group, the TG/HDL-C level in the highest group increased (OR: 1.35, 95% CI: 1.12–1.63), while the smallest OR for the highest VAI tertiles with regard to CKD was 1.26 (95% CI: 1.03–1.53). Further analysis using fitted curves confirmed the dose–response association between the four surrogate markers of IR and the prevalence of CKD and showed that the association between the four surrogate markers of IR and the prevalence of CKD was linearly positive (Figure 2). Moreover, LAP was better than the other surrogate markers of IR for predicting CKD (Supplementary Figure 3, Supplementary Table 1).

**Table 2:** Odds ratio of CKD according to continuous or tertiles of surrogate markers of IR.

**Variables**	***N***	**Events (%)**	**CKD** ***OR*** **(95%CI)**		
			**Model 1**	**Model 2**	**Model 3**
TyG	13,055	1,218 (9.33%)	1.53 (1.37, 1.70)	1.52 (1.35, 1.71)	1.42 (1.24, 1.64)
Tertiles of TyG					
T1(<8.58)	4,352	440 (10.11%)	1	1	1
T2(8.58–9.09)	4,351	383 (8.80%)	1.17 (1.01, 1.37)	1.13 (0.96, 1.34)	1.08 (0.91, 1.28)
T3(≥9.09)	4,352	395 (9.08%)	1.66 (1.42, 1.95)	1.64 (1.38, 1.95)	1.48 (1.21, 1.81)
P for trend			<0.001	<0.001	<0.001
LgLAP	13,055	1,218 (9.33%)	1.38 (1.17, 1.62)	1.51 (1.21, 1.89)	1.31 (1.03, 1.67)
Tertiles of LAP					
T1(<21.70)	4,352	509 (11.70%)	1	1	1
T2(21.70–46.64)	4,350	355 (8.16%)	1.00 (0.86, 1.16)	1.03 (0.86, 1.24)	1.02 (0.84, 1.23)
T3(≥46.64)	4,353	354 (8.13%)	1.40 (1.19, 1.64)	1.43 (1.15, 1.78)	1.34 (1.06, 1.69)
P for trend			<0.001	0.001	0.015
lgVAI	13,055	1,218 (9.33%)	2.27 (1.84, 2.79)	2.02 (1.59, 2.57)	1.67 (1.29, 2.16)
Tertiles of VAI					
T1(<1.10)	4,352	454 (10.43%)	1	1	1
T2(1.10–2.13)	4,351	393 (9.03%)	1.27 (1.09, 1.48)	1.17 (0.99, 1.39)	1.08 (0.90, 1.28)
T3(≥2.13)	4,352	371 (8.52%)	1.63 (1.38, 1.92)	1.46 (1.21, 1.76)	1.26 (1.03, 1.53)
P for trend			<0.001	<0.001	0.024
lgTG/HDL-C ratio	13,055	1,218 (9.33%)	2.43 (1.96, 3.01)	2.15 (1.68, 2.74)	1.78 (1.37, 2.31)
Tertiles of TG/HDL-C ratio					
T1(<0.71)	4,352	406 (9.33%)	1	1	1
T2(0.71–1.27)	4,351	427 (9.81%)	1.40 (1.20, 1.63)	1.28 (1.09, 1.50)	1.18 (0.99, 1.39)
T3(≥1.27)	4,352	385 (8.85%)	1.71 (1.46, 2.01)	1.52 (1.27, 1.82)	1.35 (1.12, 1.63)
P for trend			<0.001	<0.001	0.002

*Model 1 was adjusted for age, sex*.

*Model 2 was adjusted for age, sex, BMI, education, physical activity*.

*Model 3 was adjusted for age, sex, BMI, education, physical activity, current smoking, current drinking, SBP, DBP, pulse, Hcy, AST, GGT, LDL-C, Antihypertensive drugs, Antiplatelet drugs, Self-reported diabetes*.

### Subgroup Analyses by Potential Effect Modifers

A stratified analysis of potential covariates was conducted in this survey. When participants were stratified by sex (male or female), BMI (<25 or ≥25 kg/m^2^), current smoking (no or yes), current drinking (no or yes), hypertension (yes or no), physical activity (mild, moderate, vigorous), serum LDL cholesterol (<2.6 or ≥2.6 mmol/L), SBP (<140, 140–159, ≥160 mm Hg), and self-reported diabetes (no or yes), the association of four surrogate markers of IR and the prevalence of CKD remained consistent (all *P* for interactions > 0.05; see Additional file 1: Supplementary Figures 1, 2). Nevertheless, there was a significant interaction between subgroups stratified by age (<65 or ≥65 years) between TyG, VAI, and TG/HDL and the prevalence of CKD, and a strong positive correlation between TyG, VAI, and TG/HDL and the prevalence of CKD was observed in elderly individuals over 65 years old (Supplementary Figures 1, 2).

## Discussion

This large-scale cross-sectional survey among Chinese hypertensive participants ≥18 years old provides comprehensive estimates of the effects of four surrogate markers of IR on CKD in China. After adjusting for confounders, including demographic characteristics, lifestyle habits, previous medical diagnoses, drugs used, and biochemical indicators, TyG, LAP, VAI, and TG/HDL were independently and positively correlated with the prevalence of CKD. Moreover, LAP was better than the other surrogate markers of IR for predicting CKD. However, only TyG, VAI, and TG/HDL were positively correlated with the prevalence of CKD in patients over 65 years old.

In line with previous studies (25–28, 30), TyG, LAP, VAI, and TG/HDL were independently and positively correlated with the prevalence of CKD. However, in previous studies, these indicators were regarded as obesity-related indicators, and only one or two indicators were discussed in relation to CKD. Moreover, most of the above studies were conducted in healthy people and found gender differences between them. Dai et al. (27). conducted a cross-sectional study with 11,192 participants in northern China to study the relationship between two new obesity indicators (VAI and LAP) and CKD. The results showed that high levels of VAI and LAP were closely related to a higher risk of CKD but only in women, and compared with waist circumference and BMI, VAI and LAP had a better ability to predict CKD. However, studies by Seong et al. (26) found different results. A cross-sectional study based on the data from the Korean Nutrition Survey included 4,947 adults over the age of 20 and showed that CKD risk was positively correlated with the LAP index and VAI index only in men but not in women in the fully adjusted model (26). These differences in research results may be due to the different ages of the women in the study population. The women in the study by Seong et al. were mostly premenopausal women, and estrogen has an obvious protective effect on the kidney (38), so it can resist kidney damage caused by increases in LAP and VAI levels. The purpose of the second study of the China Health and Retirement Longitudinal Study (CHARLS) was to explore the relationship between TG/HDL and the risk of renal function decline. The results showed that TG/HDL was an independent risk factor for renal function decline (30). A cohort study conducted by Okamura et al. (25) included 6,026 males and 5,686 females. The aim of cohort study was to observe the influence of the TyG index on incident CKD. The conclusion of this study was that the TyG index can predict the risk of CKD in both men and women.

However, our study did not find any sex differences between the above markers of IR and CKD but rather found significant differences between TyG, VAI, and TG/HDL and CKD observed in different age groups. Compared with TyG, VAI and TG/HDL, LAP had a better ability to predict CKD. A strong positive correlation between TyG, VAI, and TG/HDL and the prevalence of CKD can be observed in elderly individuals over 65 years old. Although the LAP index was positively correlated with CKD in patients over 65 years old, its positive correlation was not significant. Age is an important factor for determining the prevalence of CKD; moreover, animal experiments show higher insulin levels with increasing age (39), so the subgroup results appear. The mechanism of CKD caused by IR can be explained from the following aspects: inflammation, oxidative stress and metabolic acidosis. First, Shimobayashi et al. (40) demonstrated that IR induced adipose tissue inflammation by inhibiting the insulin signaling pathway and increasing monocyte chemoattractant protein one production. M2 macrophages activated by adipose tissue inflammation produce and release proinflammatory cytokines, such as interleukin-6 and tumor necrosis factor-α. Inflammatory factors such as interleukin-6 and tumor necrosis factor-α can cause dysfunction of the glomerular endothelium, which leads to CKD (41, 42). Second, oxidative stress and inflammation impair the activation of nuclear factor erythroid-2-related factor-2, which protects the kidney from tissue damage (43). Finally, metabolic acidosis increases renal plasma flow and glomerular filtration rate to discharge excessive acid load, leading to impaired renal function (44).

There are also some limitations to this study. First, this is a cross-sectional investigation, and we only analyzed the correlation between four surrogate markers of IR and CKD without attempting to identify the causality or mechanisms. Second, although a large number of possible confounding factors were adjusted in the multivariate regression analysis, there may still be unidentified residual confounding variables. Third, the current study mainly focused on a Chinese hypertension population, so the associations identified here should also be examined in another population before generalization. The main advantages of this study are that it explored the relationship between four surrogate markers of IR and CKD in Chinese hypertensive patients for the first time and found that age can modify the relationship between them and that VAI and the TG/HDL-C ratio were better than the other surrogate markers of IR for predicting CKD.

## Conclusions

In summary, a substudy of the Chinese Type H Hypertension Registration Study demonstrated that four surrogate markers of IR were independently and positively correlated with CKD, and the LAP was better than the other surrogate markers of IR for predicting CKD. Only among participants aged over 65 years were higher levels of TyG, VAI and TG/HDL found to be closely related to the increased prevalence of CKD. These four surrogate markers of IR can be easily determined in the primary health care environment. Hypertensive patients with these higher indicators should receive additional screening and preventive intervention for CKD.

## Data Availability Statement

The datasets used and/or analyzed in the current study are available from the corresponding author upon reasonable request.

## Ethics Statement

The studies involving human participants were reviewed and approved by Ethics Commission of Anhui Medical University' Institute of Biomedicine (No. CH1059). The patients/participants provided their written informed consent to participate in this study.

## Author Contributions

YS participated in the literature search, data analysis, data interpretation, and wrote the manuscript. LH extracted and collected data. LH, ML, WZ, TW, LZ, and HB conceived of the study and participated in its design and coordination. PL and XC participated in the study design and provided critical revision. All authors read and approved the final manuscript.

## Funding

This work was supported by the establishment and application of big data platform for clinical and scientific research management of hypertension in Jiangxi province (20172BCB22027), the National Natural Science Foundation of China (81760049), the Jiangxi Science and Technology Innovation Platform Project (20165BCD41005), the National Key R&D Program of China (2018YFC1312902), and the Key Project of Education Department of Jiangxi Province (GJJ170013).

## Conflict of Interest

The authors declare that the research was conducted in the absence of any commercial or financial relationships that could be construed as a potential conflict of interest.

## Publisher's Note

All claims expressed in this article are solely those of the authors and do not necessarily represent those of their affiliated organizations, or those of the publisher, the editors and the reviewers. Any product that may be evaluated in this article, or claim that may be made by its manufacturer, is not guaranteed or endorsed by the publisher.

## Abbreviations

IR: Insulin resistance
CKD: chronic kidney disease
TyG: triglyceride-glucose index
LAP: lipid accumulation product index
VAI: visceral adiposity index
TG/HDL ratio: triglycerides to high-density lipoprotein cholesterol ratio
CHHRS: China H-type Hypertension Registry Study
BMI: body mass index
SBP: systolic blood pressure
DBP: diastolic blood pressure
Hcy: homocysteine
LDL-C: low-density lipoprotein cholesterol
AST: aspartate aminotransferase
ALT: alanine aminotransferase
GGT, Serum γ -glutamyltransferase: U/L.
