# Supplementary material for: Association Between the Surrogate Markers of Insulin Resistance and Chronic Kidney Disease in Chinese Hypertensive Patients
Source: Front Med (Lausanne). 2022 Feb 7;9:831648. doi: 10.3389/fmed.2022.831648 (PMC8859105; doi:10.3389/fmed.2022.831648)
Supplement: Supplementary file 1 [file Data_Sheet_1.docx]

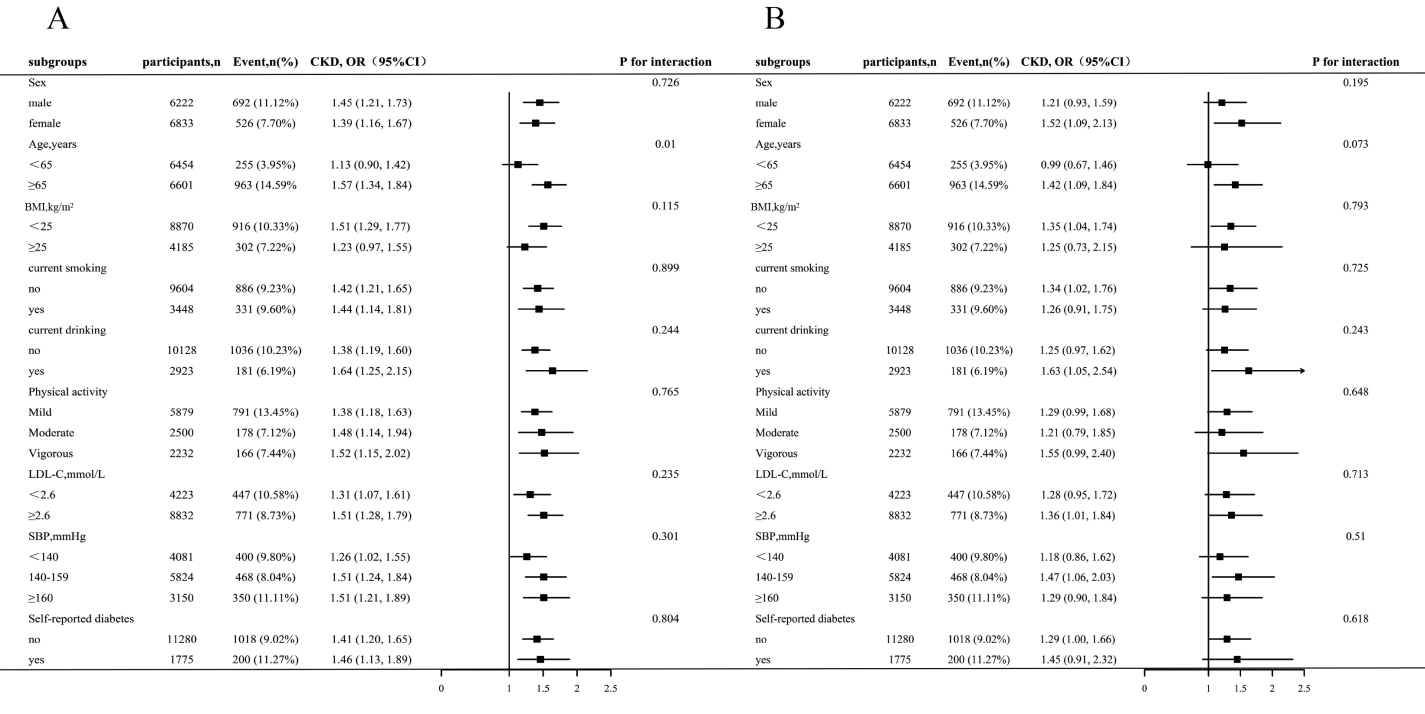


**Figure S1 The association between TyG (A), LAP (B) and the risk of CKD in various subgroups*.**

^*^Adjusted, if not stratified, for age, sex, BMI, education, physical activity, current smoking, current drinking, SBP, DBP, pulse, Hcy, AST, GGT, LDL-C, Antihypertensive drugs, Antiplatelet drugs, Self-reported diabetes.


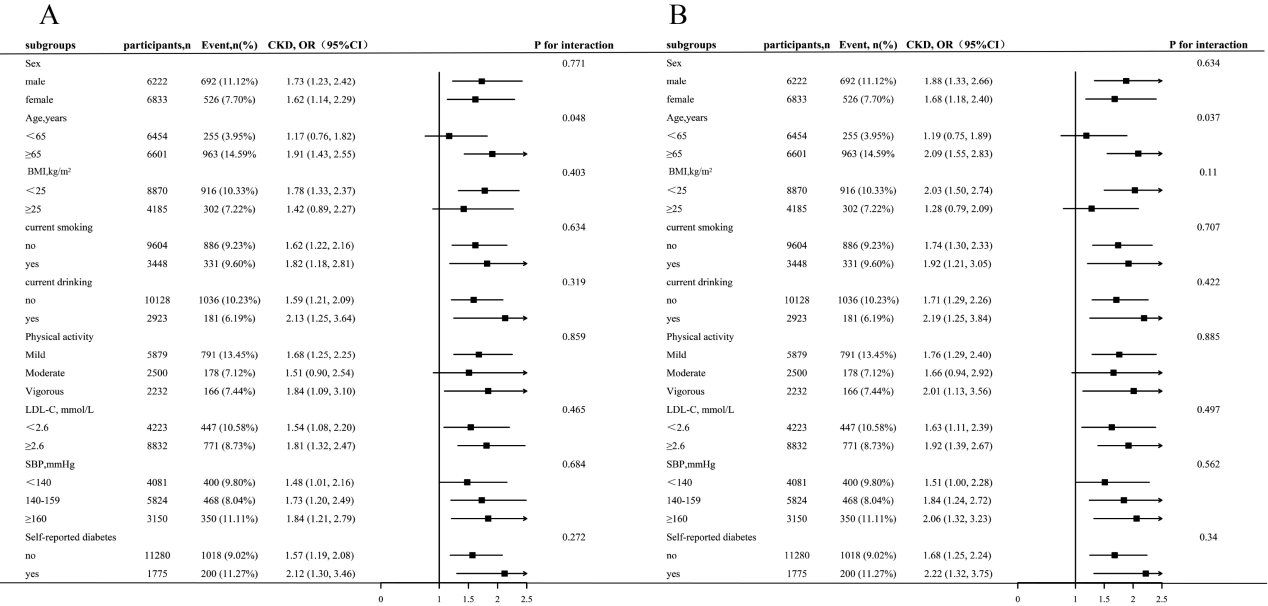


**Figure S2 The association between VAI (A), TG/HDL (B) and the risk of CKD in various subgroups*.**

^*^Adjusted, if not stratified, for age, sex, BMI, education, physical activity, current smoking, current drinking, SBP, DBP, pulse, Hcy, AST, GGT, LDL-C, Antihypertensive drugs, Antiplatelet drugs, Self-reported diabetes.


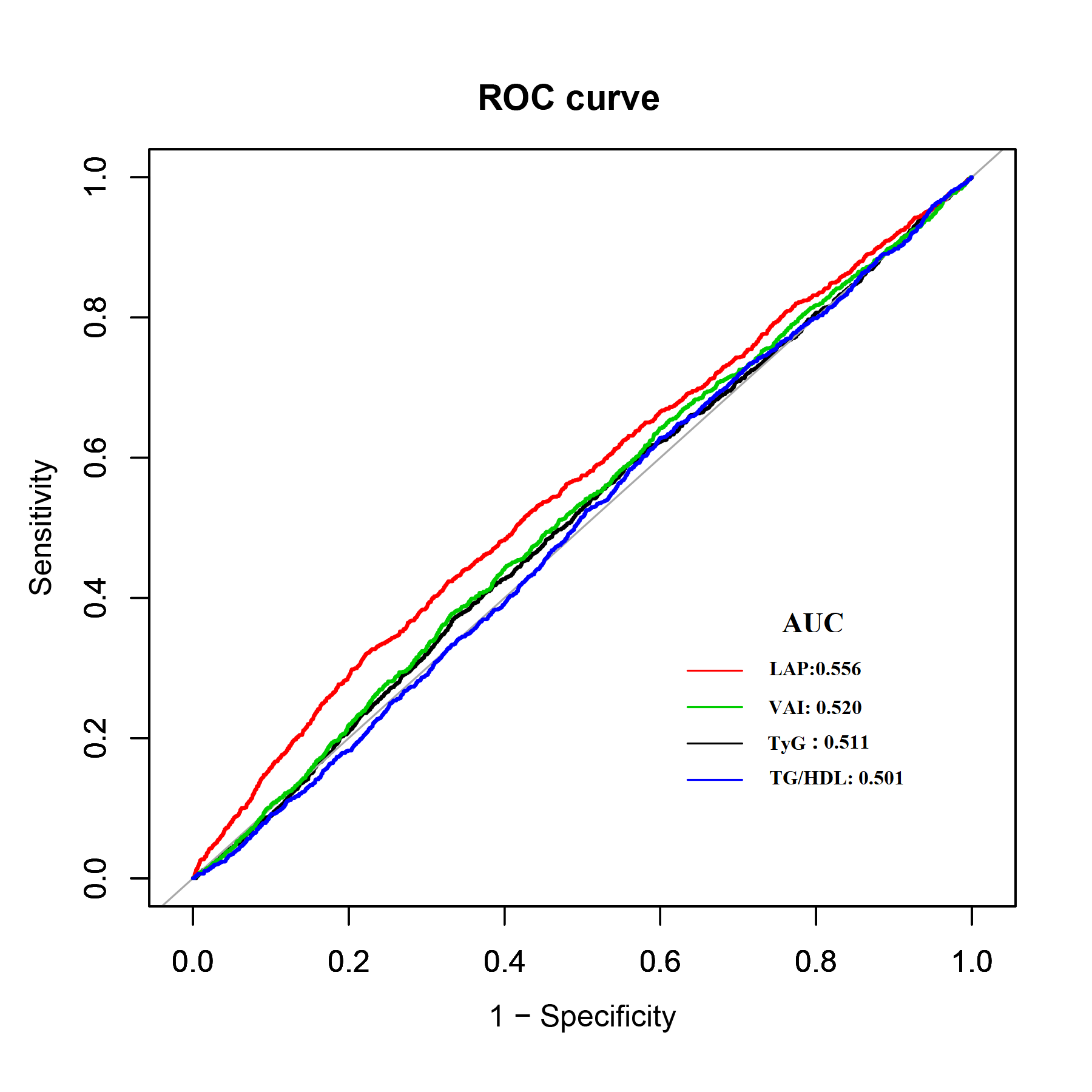


Figure S3. ROC curves for each variable for the screening of

CKD

Table S1.The area under curve, cut-off, specificities and sensitivities of each variable for the screening of CKD

| Index | AUC | 95%CI | cut-off | Specificity | Sensitivity |
| --- | --- | --- | --- | --- | --- |
| TyG | 0.511 | 0.494-0.528 | 8.588 | 0.663 | 0.373 |
| LAP | 0.556 | 0.538-0.573 | 15.746 | 0.778 | 0.320 |
| VAI | 0.520 | 0.503-0.537 | 1.108 | 0.668 | 0.377 |
| TG/HDL | 0.501 | 0.484-0.517 | 1.137 | 0.401 | 0.628 |

AUC: area under curve; CI: confidence interval; triglyceride-glucose index, LAP: lipid accumulation product; VAI: visceral adiposity index;
